# Calcined Corncob-Kaolinite Combo as New Sorbent for Sequestration of Toxic Metal Ions From Polluted Aqua Media and Desorption

**DOI:** 10.3389/fchem.2018.00273

**Published:** 2018-07-04

**Authors:** Helen O. Chukwuemeka-Okorie, Peter N. Ekemezie, Kovo G. Akpomie, Chisom S. Olikagu

**Affiliations:** ^1^Department of Pure & Industrial Chemistry, Nnamdi Azikiwe University, Awka, Nigeria; ^2^Department of Chemistry, Michael Okpara University of Agriculture, Umudike, Nigeria; ^3^Department of Pure & Industrial Chemistry, University of Nigeria, Nsukka, Nigeria

**Keywords:** abstraction, corn cob-kaolinite, composite, heavy metals, binary system

## Abstract

This study investigated a new area of improving the adsorption capacity of clay using corn cob as an alternative means of clay modification to the more expensive and complicated chemical treatment techniques. Kaolinite Clay (KC), Calcined corncob-kaolinite Combo (CCKC), and Corncob (CC) adsorbents were utilized. The adsorbents were characterized by Fourier Transform-Infrared (FT-IR) Spectroscopy, Scanning Electron Microscopy (SEM), X-ray fluorescence spectroscopy, and Brunauer-Emmett-Teller (BET) surface area analyzer. Batch adsorption methodology was used to investigate the effect of pH, initial metal concentration, adsorbent dose, and contact time on adsorption of Pb (II) and Cd (II). A slight increase in BET surface area of 29.31 m^2^/g for CCKC from 14.12 m^2^/g for raw KC was achieved. The trend of metal adsorption on the adsorbents was in the order CC>CCKC>KC. The Langmuir isotherm was found to present the best fit for the unmodified adsorbents while the Freundlich was applicable for CCKC indicating multilayer heterogeneous surface. The pseudo second order kinetic model was found to be suitable in the kinetic analysis. Thermodynamic studies revealed a spontaneous physical adsorption process of metal ions on CCKC. The combo adsorbent showed highest percentage desorption (>70%) of Cd and Pb ions in both acid and basic media compared to the other adsorbents. The results of the study established the efficiency of calcined corn cob kaolinite combo as suitable adsorbent for metal ions.

## Introduction

The pollution of water bodies with heavy metals released from industrial effluents is a serious environmental problem. The treatment of such industrial effluents is therefore very important as they may contaminate receiving water bodies (Weber et al., 1991). Industrial operations such as mining, paints and pigment manufacturing, metal plating, ceramics, glass, and battery manufacturing usually generate huge amount of effluents contaminated with heavy metals. Industrial wastewaters usually contain Cr, Ni, Zn, Cd, Pb, and Cu (Argun and Dursun, 2008). Exposure of harmful heavy metals to the eco-system leads to accumulation in humans through food chains or direct intake (Meena et al., 2008). The treatment of heavy metal contaminants in wastewaters is usually performed using activated carbon adsorption, membrane methods, filtration, solvent extraction, coagulation, evaportation, and ion exchange processes (Panayotova and Velikov, 2003). Such processes are usually limited in terms of cost and efficiency (Kurniawan et al., 2006). Adsorption is the most efficient treatment technique for heavy metals sequestration (Liang et al., 2010). Activated carbon is the adsorbent of choice in adsorption studies because it is the most effective adsorbent with a high adsorption capacity for both inorganic and organic pollutants. However, it is expensive which limits its widespread use, leading to the search of cheaper alternative adsorbents. Several researchers thus have utilized low cost adsorbents such as biomass, microorganisms, ash, and clays for heavy metals attenuation as well as other cationic and anionic contaminants from aqua media (Ahluwalia and Goyal, 2005; Meitei and Prasad, 2013; Al-Faze and Kooli, 2014; Dawodu and Akpomie, 2014; Kooli et al., 2015). Nigeria is blessed with an abundance of clay minerals which can be utilized as cheap alternative adsorbent for heavy metals sequestration. However, clay minerals especially kaolinite usually have low adsorption capacity, but the application of acid, alkaline, surfactant, and organic modification have been found to increase the adsorption capacity greatly (Bhattacharyya and Gupta, 2006; Unuabonah et al., 2008; Akpomie and Dawodu, 2016). However, the use of these chemicals for modification is expensive; require skilled personnel and results in secondary contamination. Therefore, it is necessary to look for other low cost alternative means of modifying clay in order to improve their adsorption capacity. The active sites of an adsorbent are known to affect the adsorption capacity of the material significantly; hence the addition of biomass materials to clay could increase the surface area, provide more efficient active sites and thus enhance the adsorption capacity of the combo adsorbent. The modification of clay with rice husk was found to improve the adsorption capacity for heavy metals (Akpomie and Dawodu, 2015). Corn cob has been reported to have high adsorption capacity for heavy metals (Arunkumar et al., 2014) and could possibly be used in clay modification to enhance the adsorption. However, despite the high adsorption capacity, it has not been used to modify clay. This study therefore exploits the potential of corn cob-kaolinite combo as a novel adsorbent for attenuation of Pb (II) and Cd (II) ions from polluted solution.

## Materials and methods

### Adsorbent preparation procedure and characterization

The kaolinite clay was obtained from Aloji in Kogi state, Nigeria and prepared as described by Dawodu and Akpomie (2014) to obtain the Kaolinite Clay (KC) adsorbent. The corn cob was obtained from Emene, Enugu, Nigeria. Thereafter it was washed with de-ionized water to get rid of unwanted materials attached to the surface. The corn cob was then sundried for 7 days after which it was pulverized. The samples were then passed through 100 μm mesh screen, obtaining the Corn Cob (CC) adsorbent. The modification of clay with corn cob was performed as described; the raw corn cob was added to the kaolinite clay at a percentage composition of 10, 20, 30, 40, and 50 by weight. The mixture was compounded with a mortar and pestle after addition of distilled water to aid proper mixing. It was then sundried, pulverized and heated in a muffle furnace at 300°C and passed through 100 μm mesh sieve to obtain the Corn Cob-kaolinite composite (CCKC) adsorbent.

The chemical oxide composition of KC and CCKC was determined by the X-ray fluorescence spectrometer. The pH at point of zero charge of the adsorbents was obtained as previously described (El-Shafey et al., 2012). Surface area and pore characteristics were obtained using the micrometrics ASAP 2010 model BET analyzer. The surface functional groups on the adsorbents responsible for binding of metal ions were determined by the Fourier-Transform Infrared (FTIR) spectrophotometer (Shimadzu FT-IR 8400s). Adsorbents morphology was determined by the Scanning Electron Microscope (SEM, Hitachi S4800 model).

### Adsorbate preparation, batch adsorption, and desorption studies

The entire chemicals used were of analytical grade and were not purified further. A laboratory solution of lead (II) and Cadmium (II) ions were prepared by dissolving appropriate amounts of Pb(NO_3_)_2_ and Cd(NO_3_)_2_, respectively in 50 mL of de-ionized water in a beaker. The salt solution was stirred with a glass rod until completely soluble. Thereafter, the solution was then placed in a 1 L volumetric flask, which was made up to the meniscus mark with de-ionized water to obtain a stock solution of concentration 1,000 mg/L of the metal ions. Serial dilution was utilized to prepare lower concentrations which include 200, 400, 600, and 800 mg/L from the stock solution.

Batch adsorption procedure was carried out in 100 mL plastic bottles by contacting 0.5 g of adsorbent with 50 mL of metal solution. The effect of pH (2.0–8.0), initial metal ion concentration (200–1,000 mg/L), adsorbent dose (0.5–2.5 g), contact time (5–120 min), and temperature (300–323 K) were investigated by varying the factors in their given range. The effect of pH was performed at a metal concentration 600 mg/L, time 120 min, and temperature 300 K. Influence of initial metal concentration was tested at a pH 6.0, time 120 min, and temperature 300 K while adsorbent dose was performed under similar conditions using metal concentration of 600 mg/L. Contact time effect was carried out at pH 6.0, metal concentration 600 mg/L, and temperature 300 K. A thermo-stated temperature water bath was used to study the effect of temperature by varying the temperature at constant pH of solution 6.0, time 120 min, and metal concentration of 600 mg/L. At various time intervals, samples were collected from the bottles and analyzed for residual metal ions using the Atomic Absorption Spectrophotometer (AAS) (Buck Scientific model 210VGP). The amount of metal ions adsorbed were calculated from the mass balance equation described previously (Ravishankar et al., 2016).

Desorption experiment was conducted by contacting 0.2 g of metal loaded adsorbent with 50 mL of 0.1 M HCl in 120 mL bottles. The solution was agitated for 60 min then filtered and the concentration of metal ions desorbed in filtrate was checked by the AAS. The procedure was repeated using 0.1M NaOH as desorbing agent. The percentage desorption was then calculated from the equation:

(1) $$\begin{array}{r} \%Desorption=100\left[C_{D}V_{D}\right]/q_{e}m \end{array}$$

Where *C*_*D*_ (mg/L) is the metal ion concentration desorbed, *V*_*D*_ (L) is the volume of eluent used for desorption, m (g) is the mass of adsorbent used for desorption, and *q*_*e*_ (mg/g) is the adsorption capacity of the adsorbent for metal ions obtained under optimum adsorption conditions.

### Isotherm, kinetic, and thermodynamic modeling

Isotherm modeling of was performed by the Langmuir, Freundlich, Tempkin, and Dubinin-Radushkevich (D-R) models (Ojo et al., 2017). The linear form of these model equations are presented respectively as:

(2) $$\begin{array}{rcl} C_{e}/q_{e} & = & 1/q_{L}k_{L}+C_{e}/q_{L} \end{array}$$

(3) $$\begin{array}{rcl} logq_{e} & = & logk_{F}+\left[1/n\right]logC_{e} \end{array}$$

(4) $$\begin{array}{rcl} q_{e} & = & BlnA+BlnC_{e} \end{array}$$

(5) $$\begin{array}{rcl} lnq_{e} & = & lnq_{m}-\beta {£}^{2} \end{array}$$

Where *C*_e_ (mg/L) is the equilibrium sorption concentration, *q*_e_ (mg/g) is the sorption capacity, *q*_L_ (mg/g) is the Langmuir monolayer uptake capacity, k_L_ (L/mg) is the Langmuir constant, *k*_F_ (L/g) is the Freundlich constant, *n* corresponds to the adsorption intensity, *B* (mg/g) relates to the heat of sorption, A the equilibrium binding constant, *q*_m_ (mg/g) the D-R sorption capacity, β (mol^2^/J^2^) corresponds to the free energy of sorption and £ (kJ/mol) the Polanyi potential (Lingamdinne et al., 2017; Ojo et al., 2017).

Kinetic modeling was analyzed using the linear form of pseudo first order (PFO), pseudo second order (PSO), liquid film diffusion (LFD), and intraparticle diffusion (ID) rate equations given respectively;

(6) $$\begin{array}{r} log\left(q_{e}-q_{t}\right)=logq_{e}-\left(K_{I}/2.303\right)t \end{array}$$

(7) $$\begin{array}{r} t/q_{t}=1/K_{2}q_{e}^{2}+t/q_{e} \end{array}$$

(8) $$\begin{array}{r} q_{t}=K_{d}t^{1/2}+C \end{array}$$

(9) $$\begin{array}{r} ln\left(1-F\right)=-K_{fd}t+Y \end{array}$$

Where *q*_t_ (mg/g) is the sorption capacity at time *t* (min), *K*_I_ (min^−1^) is the PFO rate constant, *K*_2_ (g/mg/min) the PSO rate constant, initial sorption rate h = *K*_2_*q*${}_{\text{e}}^{2}$ (mg/g/min), *K*_d_ (mg/gmin^1/2^) the ID rate constant with intercept C and *K*_fd_ (mg/gmin) is the LFD constant with intercept Y (Ravishankar et al., 2016; Islam et al., 2017).

Thermodynamics was evaluated to determine the standard free energy *(*Δ*G*^0^*)*, entropy change *(*Δ*S*^0^*)* and enthalpy change *(*Δ*H*^0^*)* by the use of the following equations:

(10) $$\begin{array}{r} \Delta G^{0}=-RTlnK_{c} \end{array}$$

(11) $$\begin{array}{r} lnK_{c}=-\left(\Delta H^{0}/RT\right)+\left(\Delta S^{0}/R\right) \end{array}$$

Where *K*_c_, *T (K)*, and *R* (J/mol K) represents the distribution coefficient, absolute temperature, and ideal gas constant, respectively (Song et al., 2016).

## Result and discussion

### Selection of optimum corn cob-kaolinite combo

**Figure 1:**
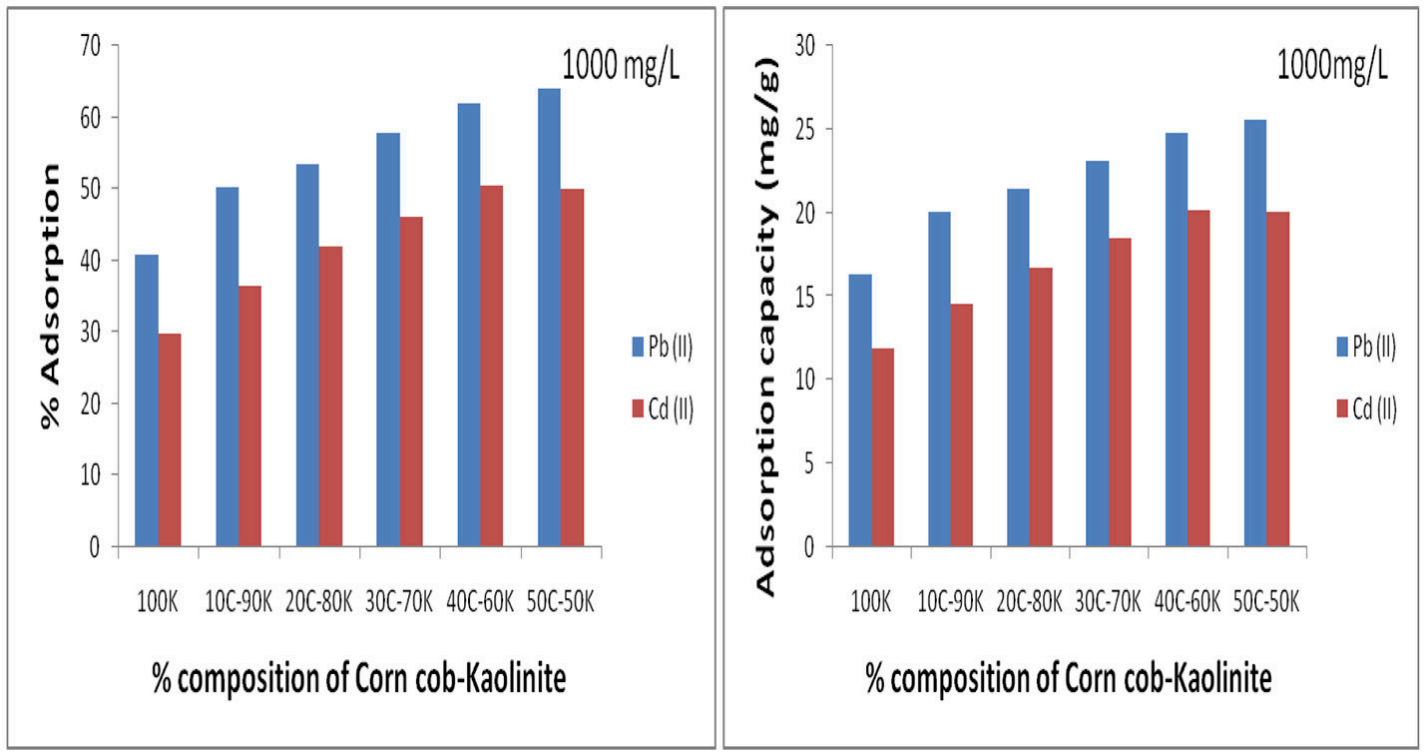
Optimization of corn cob addition to kaolinite and calcination on the adsorption of Pb (II) and Cd (II) from solution (pH 6.0, dosage 0.5 g, Contact time 180 min).

In order to select the appropriate mixing ratio for kaolinite and corn cob, five combos were prepared which comprises of 10% corn cob and 90% kaolinite (10C−90K), 20% corn cob and 80% kaolinite (20C−80K), 30% corn cob and 70% kaolinite (30C−70K), 40% corn cob and 60% kaolinite (40C−60K) and 50% each of corn cob and kaolinite (50C−50K). The adsorbents were utilized for the adsorption of the metal ions from the binary solution at metal concentration 1,000 mg/L as shown in Figure 1. It was observed that an increase in percentage adsorption and adsorption capacity of the metal ions with increase in the amount of corn cob in the composite was obtained. However, 40C−60K recording a slightly higher concentration (20.2 mg/g and 50.5%) than 50C−50K (19.3 mg/g and 48.2%) for Cd (II) ions. In general, the increase in metal adsorption with CC concentration is simply due to increase in the number of active binding sites on the kaolinite and increase in the heterogeneous nature of the adsorbent. The 40C−60K adsorbent was then chosen and utilized in this study for adsorption due to its relatively higher adsorption potential recorded than the other composites.

### Adsorbents characterization

The characterization of the three adsorbents is presented in Table 1. The oxide analysis indicated that silica (SiO_2_) and alumina (Al_2_O_3_) are the major component of KC and CCKC which also contains other metallic oxides in smaller amounts. A decrease in the metallic oxide components of CCKC compared to KC was observed due to the presence of calcined corn cob components in the former. However, the percentage Loss on ignition (LOI) of CCKC was higher than that of KC due to the burning off of the corn cob component in the composite. Similar report was observed previously (Akpomie and Dawodu, 2015). The BET surface area (S_BET_), Average pore diameter (APD) and Total pore volume (TPV) of CC was found to be higher than those of KC and CC, which might indicate a higher adsorption potential of CC. It was observed that despite the calcinations of kaolinite with corn cob, the S_BET_ of CCKC did not increase significantly. This was probably due to the impregnation of corn cob carbon during calcinations in the pores or crevices of the kaolinite (Olu-owolabi et al., 2017) which resulted in only a slight increase in S_BET_ than expected. The high pHpzc of CCKC suggests that this adsorbent would be effective over a wide range of pH (< 6.0) in the removal of anionic pollutants from solution.

**Table 1:** physicochemical characterization of the adsorbents.

**Parameter**	**KC**	**CCKC**	**CC**
SiO_2_ (%)	52.63	38.38	–
Al_2_O_3_ (%)	26.42	15.47	–
Fe_2_O_3_ (%)	0.81	0.41	–
CaO (%)	0.86	0.52	–
K_2_O (%)	1.52	1.13	–
Na_2_O (%)	0.74	0.61	–
MgO (%)	0.51	0.27	–
TiO_2_ (%)	0.23	0.13	–
MnO (%)	0.34	0.17	–
LOI (%)	15.61	28.58	–
S_BET_ (m^2^/g)	14.12	29.31	93.46
APD (Å)	12.29	18.69	24.69
TPV (cm^3^/g)	0.0043	0.0137	0.0577
pH_pzc_	5.3	6.0	4.8

**Figure 2:**
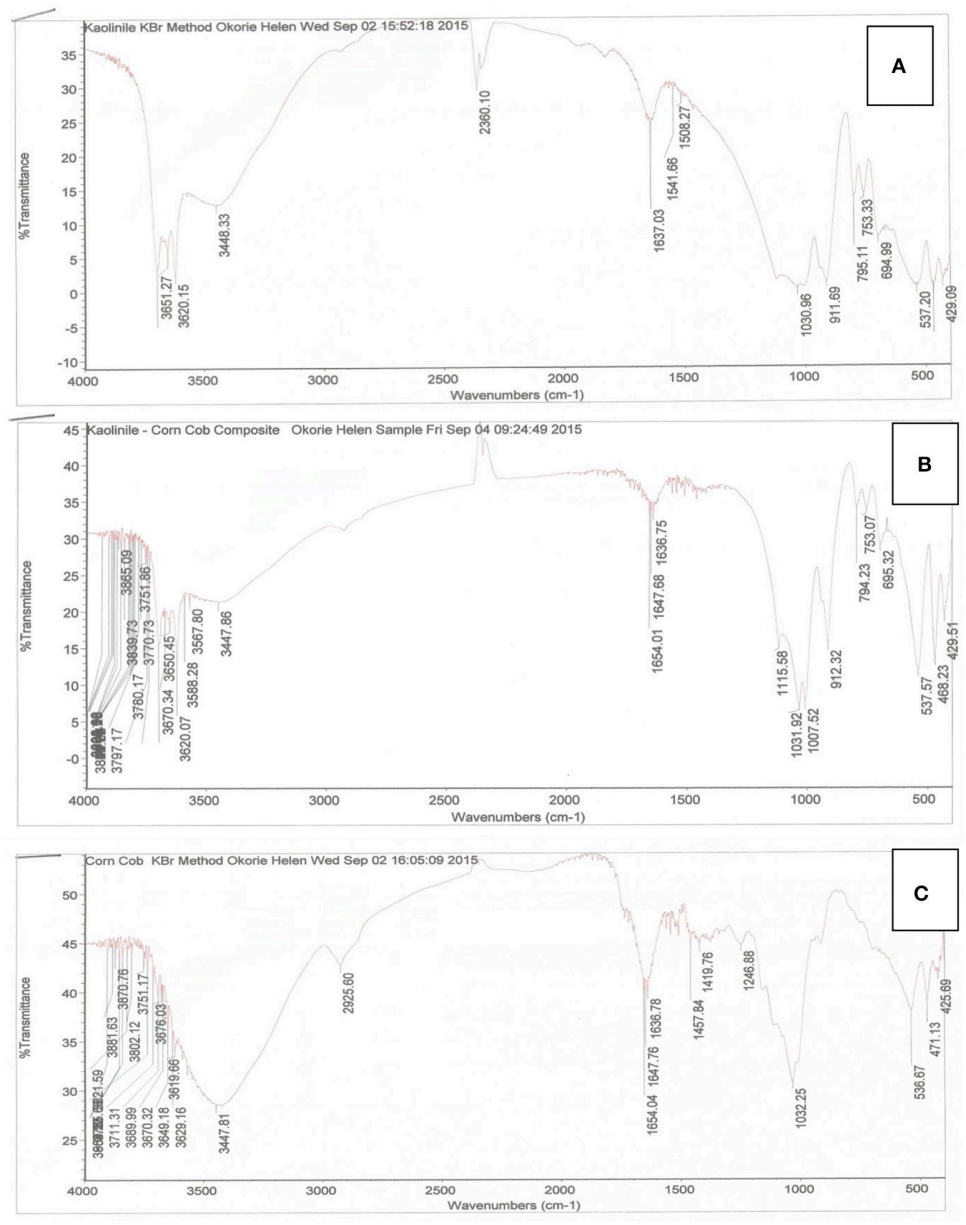
Fourier Transform infrared spectra of **(A)** kaolinite clay, **(B)** corn cob kaolinite composite, and **(C)** corn cob.

**Figure 3:**
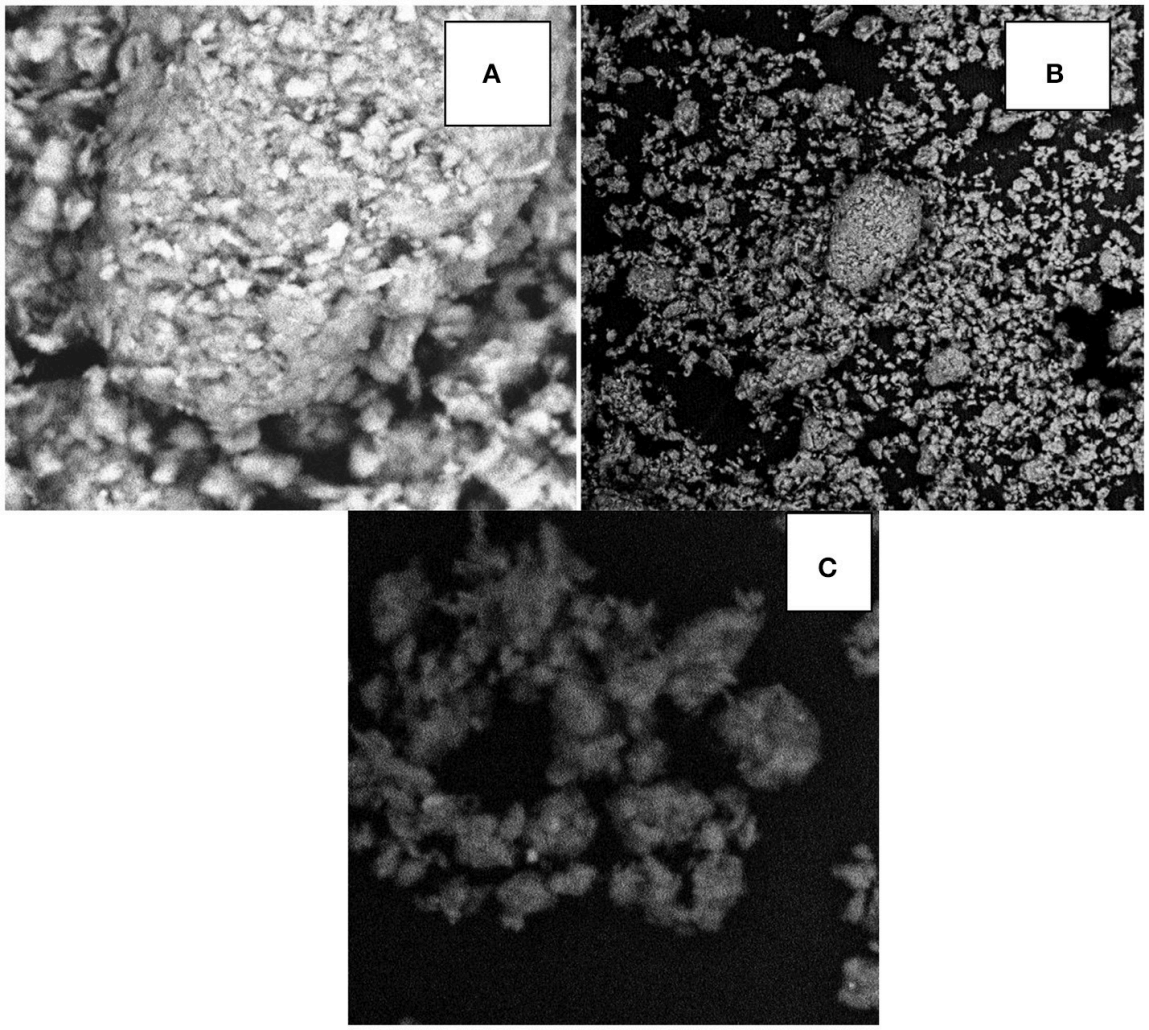
Scanning electron microscope images of **(A)** kaolinite clay, **(B)** corn cob kaolinite composite, and **(C)** corn cob.

The adsorbents were characterizedto determine the surface nature and functional groups responsible for metal adsorption by the FTIR and SEM analysis. The spectra illustrating the characterization of the adsorbents are shown in Figures 2, 3. The FT-IR is effective in the direct identification of functional groups on adsorbent surfaces (Badmus et al., 2007). Examination of the surface of adsorbents before and after modification gives useful information on the surface groups involved in the modification and confirms the modification of the adsorbent. The FT-IR spectrum of KC (Figure 2) showed several bands, which suggests the presence of functional groups on the kaolinite. Absorption bands (3651.27, 3620.15 cm^−1^) and 3448.33 cm^−1^ corresponds to the inner and outer OH stretching vibrations of kaolinite respectively. 2360.18 cm^−1^ represents the P-OH stretching, while that at 1637.03 cm^−1^ is due to the OH bending of water and can also be attributed to the COO symmetric stretching (Li et al., 2011). The Si-O bending vibration was observed at 1030.96 cm^−1^ and the stretching at 753.33 and 694.99 cm^−1^. Absorption at 911.69 cm^−1^ corresponds to the Al-O bending of kaolinite. The FTIR spectrum of CCKC (Figure 2) showed that changes in the absorption bands of the kaolinite after corn cob modification was observed in the spectrum which is an indication of the successful modification of the adsorbent. Several bands were indicated in the region 3670.34 and 3447.86 cm^−1^ which suggests more –OH sites (Pathania et al., 2017) on CCKC, acquired from the CC component. The symmetric –COO- stretching or OH bending of water was also observed by bands at 1636.75–1654.01 cm^−1^. The Si-O bending vibration was observed at 1007.52–1115.58 cm^−1^. There were shifts in bands of the Si-O stretching vibration of the kaolinite from 795.11 to 794.23 cm^−1^ and from 694.99 to 695.32 cm^−1^. Also, the Al-O bending was observed at 912.32 cm^−1^. The FTIR spectrum of CC (Figure 2) showed several OH bands at 3619.66–3447.81 cm^−1^, the broad and intense band at 3447.81 cm^−1^ indicates more active OH sites on CC. This must have been responsible for the higher uptake compared to KC and CCKC as would be seen later in this study. Absorption bands at 2925.60 cm^−1^ is attributed to the CH_3_ stretching vibration of aliphatic organic compounds while bands at 1636.78–1654.04 cm^−1^ corresponds to the C = O of esters (Nwadiogbu et al., 2014). The intense nature of the C = O band also suggests high presence of this group which also might have influence the higher adsorption of CC for metal ions when compared to the other adsorbents. The C = C of alkenes was also indicated by bands at 1419.76 and 1457.84 cm^−1^, while the C = O stretching of acetyl group at 1246.88 cm^−1^. The C-O stretching vibration of primary alcohol was also observed at 1032.25 cm^−1^. However, CC did not show clay absorption bands at 690 to 915 cm^−1^ which were present in KC and CCKC attributed to the Si-O and Al-O vibration of clays. However, the spectra of the three adsorbents all showed the presence of surface functional groups responsible for the adsorption of Pb (II) and Cd (II) from aqueous media (Deniz and Ersanli, 2017).

SEM images of the adsorbents are shown in Figure 3. The SEM is used to examine the surface morphology and the porous nature of the material which influences adsorption. From the SEM images, it is observed that the adsorbents revealed a porous nature, considerable number of heterogeneous pores, an irregular surface and particle aggregation of various shapes and sizes (Meitei and Prasad, 2013). The presence of pores helps in the diffusion of metal ions into the adsorbents during the sorption of metal ions from solution. Most importantly it is seen that an increase in porosity of CCKC was observed in the SEM images when compared to KC and may suggest higher adsorption of metal ions by CCKC than KC. Also, the SEM images of KC and CCKC showed some white clumps or particles on the surface, these were likely due to the presence of non-clay minerals like potassium, iron, magnesium, sodium, calcium, and manganese (Njoya et al., 2006; Unuabonah et al., 2008). Also, it is observed from the SEM images that CC adsorbent was more porous than KC and CCKC which may have accounted for the higher adsorption of CC than the other adsorbents as seen later. In general, the porous nature of the adsorbents is desirable for efficient adsorption of metal ions from solution.

### Influence of solution pH

**Figure 4:**
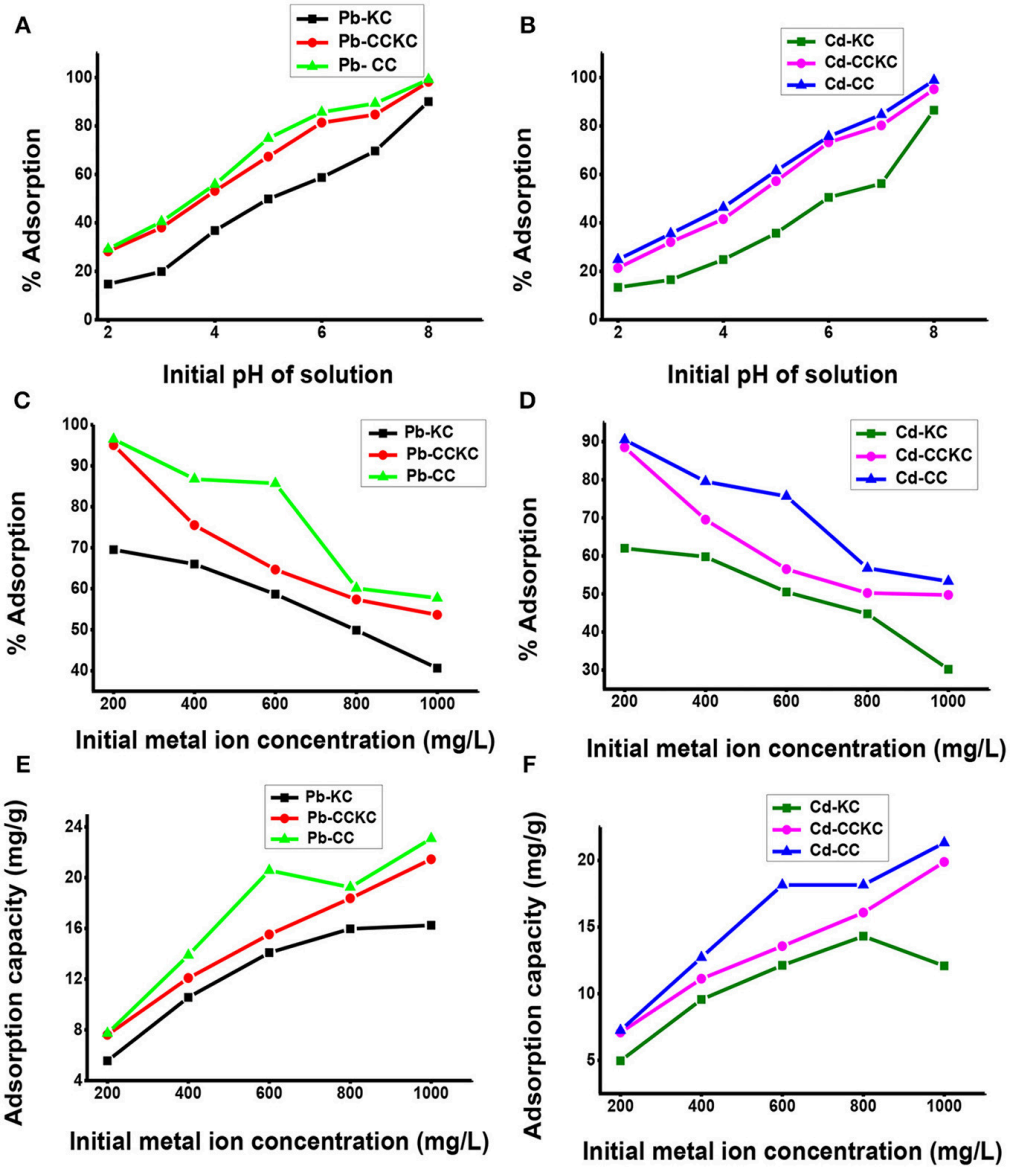
Effect of pH on the adsorption of **(A)** lead **(B)** cadmium, effect of initial metal ion concentration on the percentage adsorption of **(C)** lead **(D)** cadmium and adsorption capacity of the adsorbents for **(E)** lead and **(F)** cadmium ions.

The initial solution pH is important in adsorption since it affects adsorbate degree of ionization and speciation and adsorbent surface charge (Meitei and Prasad, 2013). The influence of pH on the sequestration of Pb (II) and Cd (II) ions on the adsorbents is shown in Figure 4. The percentage adsorption of both metal ions on all the adsorbents showed an increase with increase in initial pH of solution. With an increase in initial solution pH from 2.0 to 8.0, the percentage adsorption of Pb (II) increased from 14.67 to 90, 28.17 to 98.17, and 29.17 to 99.17%, for KC, CCKC, and CC respectively. Similarly, with increase in the initial solution pH from 2.0 to 8.0, the adsorption of Cd (II) showed a percentage increase from 13.33 to 86.5, 21.33 to 95.17, and 24.83 to 98.83 for the respective adsorbents. The low adsorption recorded at lower pH values is simply due to excess H^+^ ions in solution which competes with the metal ions for the active sites of the adsorbent, resulting in low removal (Arunkumar et al., 2014). As pH increased, the H^+^ ions in solution decreased, this reduced the competition between the metals and H^+^ ions for the active sites of the adsorbents leading to an increase in percentage adsorption (Gueye et al., 2014). Furthermore, for pH values greater than 6.0, there is metal precipitation from solution in the form of hydroxides and this usually accounts for the higher removal recorded at pH 7.0 and 8.0 (Taffarel and Rubio, 2009). The pH value of 6.0 was utilized in this research and not 8.0, despite the highest removal obtained at 8.0. This was done in order to prevent precipitation of metal associated with higher pH values (7.0–8.0) and ensure that adsorption phenomena accounts for the optimum adsorption recorded at 6.0 and not precipitation.

Most importantly, in comparing the adsorption potential of the three adsorbents for metal ions, it is found that for all pH values for both Pb (II) and Cd (II) ions, the adsorption followed the order CC>CCKC>KC. This result clearly indicated that the addition of CC to KC and calcination improved the adsorption of the clay which is desired in this study. Although, CC still gave a slightly higher adsorption than CCKC, this suggests the use of CC in modifying clay and not the use of KC for modifying CC for metal adsorption. This is expected since biomass materials usually have higher adsorption potentials than clay minerals. Similar result on improving the adsorption capacity of a montmorillonite clay using rice husk has been reported (Akpomie and Dawodu, 2015). The higher adsorption capacity of CCKC than KC is due to increase surface area and more efficient active sites on the combo adsorbent. The composite provided different types of actives sites (due to mixing of two different materials) which may favor metal ions removal. Comparing adsorption of metals at all pH values on all the adsorbents, the following trend was observed Pb (II)> Cd (II). The difference in the adsorption trend of Pb (II) and Cd (II) may be attributed to differences in behavior of metals ions in solution. These include the smaller hydrated radius of Pb (II) (0.401 nm) compared to Cd (II) (0.426 nm), the higher electronegativity of Pb (II) (2.10) than Cd (II) (1.69), whereas Pb (II) is adsorbed as hydrolysed species Cd (II) is not (Barka et al., 2013). These factors accounts for the higher adsorption of lead than cadmium on adsorbents. Similar result has been reported (Nessim et al., 2014).

### Influence of metal concentration

The influence of initialmetal concentration on the percentage adsorption of Pb (II) and Cd (II) on the adsorbents is shown in Figure 4. Decrease in percentage removal the metal ions on the adsorbents with increase in initial metal ion concentration was observed. In fact, with increase in initial concentration from 200 to 1,000 mg/L, the percentage removal of Pb (II) decreased from 69.5 to 40.60, 95.0 to 53.6, and 96 to 57.7% for KC, CCKC, and CC respectively. Similarly, the percentage adsorption of Cd (II) also showed a decrease from 62 to 30, 88.5 to 49.7, and 90.5 to 53.3% for the respective adsorbents. This decrease in percentage adsorption with increase in metal ion concentration is due to the fact that at lower concentrations, more of the metal ions would be removed by the abundant active sites on the adsorbent. Less metal ions was adsorbed at higher concentration due to saturation of adsorbent active sites (Nuhoglu and Malkoc, 2009). This implies that if the concentration is significantly increased further, a corresponding decrease in percentage removal would be expected due to complete saturation of the active sites. Similar result has been reported (Das and Mondal, 2011).

The influence of metal concentration on the adsorption uptake potential of the adsorbents for Pb (II) and Cd (II) was also determined as illustrated in Figure 4. Unlike the decrease observed in percentage adsorption, an increase in adsorption capacity for the ions on all the adsorbents with increase concentration was recorded. With increase in metal ion concentration from 200 to 1,000 mg/L, an increase in the sorption uptake of Pb (II) from 5.56 to 16.24, 7.6 to 21.44, and 7.72 to 23.08 mg/g and Cd (II) from 4.96 to 12.08, 7.08 to 19.88, and 7.24 to 21.32 mg/g for KC, CCKC, and CC was obtained respectively. The increase in uptake capacity with initial ion concentration observed is due to increased concentration, which generated a driving force, overcoming mass transfer resistance between aqueous and solid phase (Kumar and Tamilarasan, 2013). Higher concentration in solution simply implies more metal ions available on the adsorbent surface and maximum utilization of the active sites (Barka et al., 2013). Similar result has been reported (Karthikeyan et al., 2004). The adsorption of metal ions on the adsorbents at all concentrations also followed the order CC>CCKC>KC, while the metal ions adsorped by the adsorbents followed the trend Pb>Cd. The metal concentration of 600 mg/L was utilized in this study and not 200 mg/L despite the high percentage removal at 200 mg/L. This was done so as to be able to study the effect of adsorbent dose effectively to avoid all the metals at low concentrations of 200 mg/L to be adsorbed at higher adsorbent doses.

### Isotherm modeling

Equilibrium isotherm data provides information on surface properties of adsorbents, adsorption mechanism and sorbents affinity (Nuhoglu and Malkoc, 2009; Das and Mondal, 2011). The Langmuir, Freundlich, Tempkin, and Dubinin-Radushkevich (D-R) isotherm models were applied in the analysis of the equilibrium sorption data. The most suitable isotherm is determined by the linear regression coefficient (*R*^2^). The *R*^2^ value closest to one indicates the best fit model. The equilibrium isotherm model constants for the adsorption of Pb (II) and Cd (II) ions unto the adsorbents are shown in Table 2. The Langmuir correlation coefficient (*R*^2^) of Pb (II) and Cd (II) on KC and CC were very high (close to 1), indicating a good fit of the Langmuir isotherm model in the description of the sorption process on these adsorbents. This indicated that the surfaces of KC and CC are homogenous in nature (the active sites on the surface of each adsorbent are identical) and involves a monolayer sorption of Pb (II) and Cd (II) ions. This homogenous nature is supported by the fact that these adsorbents were not mixed together like CCKC, thereby retaining their individual homogenous nature of active sites. The maximum monolayer adsorption capacity for Pb (II) adsorption was 20.83, 23.26, and 23.26 mg/g, while that for Cd (II) was 14.71, 21.74, and 22.73 mg/g for KC, CCKC, and CC respectively. This again showed the best adsorption for CC followed by CCKC and the least for KC, and also a higher adsorption of Pb (II) than Cd (II) ions on the adsorbents as discussed earlier. An important Langmuir separation factor (*R*_*L*_) is given by the relationship (Akpomie and Dawodu, 2016):

(12) $$\begin{array}{r} R_{L}=1/\left[1+K_{L}Co\right] \end{array}$$

*R*_*L*_ gives useful information on the adsorption nature. The adsorption is linear (*R*_*L*_ = 1), irreversible (*R*_*L*_ = 0), unfavorable (*R*_*L*_> 1), or favorable (0 < *R*_*L*_ < 1). For concentrations from 200 to 1000 mg/L for the two metal ions on all the adsorbents, *R*_*L*_ values ranged from 0.023 to 0.417 for Pb(II) and 0.05–0.333 for Cd(II) which showed a favorable sorption of the metals on all the adsorbents, showing their usefulness in the removal of Pb (II) and Cd (II) from contaminated solutions. The Freundlich values of the correlation coefficient (*R*^2^) for Pb (II) and Cd (II) ions are lower than those of the Langmuir isotherm model for KC and CC. However, the *R*^2^ values obtained for CCKC of 0.973 and 0.967 for Pb (II) and Cd (II) ions, respectively, are higher than those of all the other models. This implies that the Freundlich isotherm presented the best fit for the adsorption process on CCKC. This indicates a heterogeneous surface of CCKC involving multilayer adsorption of metal ions, rather than a monolayer homogenous adsorption. The heterogeneous surface of CCKC is expected due to the mixing of KC and CC together providing different types of active sites on the surface of CCKC. Values of *n* between 1 and 10 also depict a favorable adsorption process (Yadav et al., 2014). The value of *n* obtained for the adsorption of Pb (II) and Cd (II) ions are within the range of 2.11–3.97 and 2.35–3.06 indicating again a favorable adsorption of metal ions by the adsorbents. The linear regression coefficient obtained from the Temkin model for both metal ions on all the adsorbents were lower than the Langmuir and Freundlich isotherms. It can be concluded that the Temkin isotherm did not provide good fit to the adsorption as the Langmuir isotherm for the adsorption of metal ions on KC and CC and the Freundlich isotherm for metal adsorption on CCKC. This isotherm is therefore not suitable in the description of the adsorption mechanism of metal ions on the adsorbents. Again, looking at the values of the linear regression coefficient (*R*^2^) of the D-R isotherm, it is observed that this model did not give good fits compared to the Langmuir and Freundlich isotherms. The only exception was noted in the adsorption of Cd (II) ions on KC in which the R^2^ value of 0.960 presented by the D-R isotherms was better than those of all the models discussed previously.

**Table 2:** Equilibrium isotherm constants for the adsorption of Pb (II) and Cd (II) on the adsorbents.

		**Pb (II)**			**Cd (II)**	
**Isotherm/Adsorbent**	**KC**	**CCKC**	**CC**	**KC**	**CCKC**	**CC**
**LANGMUIR**						
q_L_ (mg/g)	20.83	23.26	23.26	14.71	21.74	22.73
K_L_ (L/mg)	0.007	0.015	0.043	0.011	0.010	0.019
*R*^2^	0.987	0.962	0.981	0.945	0.937	0.985
**FREUNDLICH**						
K_F_	1.107	3.99	5.140	0.938	2.576	2.944
1/n	0.475	0.26	0.252	0.426	0.311	0.327
N	2.11	3.85	3.97	2.35	3.22	3.06
*R*^2^	0.910	0.973	0.878	0.789	0.967	0.933
**TEMPKIN**						
A (L/g)	0.059	0.708	1.543	0.069	0.233	0.304
B (mg/g)	4.885	3.334	3.469	3.671	3.675	4.222
R^2^	0.961	0.902	0.862	0.793	0.888	0.937
**D-R**						
qm (mg/g)	15.26	16.54	19.01	12.95	14.98	17.65
B (mol^2^/J^2^)	0	0.1 × 10^−4^	0.8 × 10^−5^	0	0.7 × 10^−4^	0.6 × 10^−4^
*R*^2^	0.955	0.734	0.829	0.960	0.728	0.845

The monolayer maximum uptake of Pb (II) and Cd (II) ions by the adsorbents was compared with other reported adsorbents as shown in Table 3. Although, our prepared adsorbents recorded quite lower adsorption compared to most sorbents especially for Pb (II) ions, their adsorption capacity for Cd (II) was quite comparable. This indicates the suitability of our prepared adsorbents for metals removal especially for Cd (II) ions.

**Table 3:** Comparison of maximum uptake capacity of the adsorbents for lead (II) and Cd (II) ions with other adsorbents in literature.

**Adsorbent**	**Pb (II) (mg/g)**	**Cd (II) (mg/g)**	**References**
Activated carbon	294.11	178.5	Karnib et al., 2014
*Ulva fasciata*	80.7	43.1	Nessim et al., 2014
*Sargassum* sp.	119.1	79.4	Nessim et al., 2014
Cactus cladodes	98.62	30.42	Barka et al., 2013
Spent gram	35.50	17.30	Li et al., 2010
Alginate	58.02	30.91	Mata et al., 2009
*Flammulina velutipes*	18.34	8.43	Zhang et al., 2010
*Launea arborescens*	129.90	11.50	Benhima et al., 2008
*Senecio anthophorbium*	149.6	18.9	Benhima et al., 2008
Wheat straw	–	14.61	Dang et al., 2009
Activated carbon	51.80	–	Mohan et al., 2017
Pine cone activated carbon	27.53	–	Mohan et al., 2017
Kaolinite clay	20.83	14.71	This work
Corn cob-kaolinite clay	23.26	21.74	This work
Corn cob	23.26	22.73	This work

### Influence of adsorbent dosage

**Figure 5:**
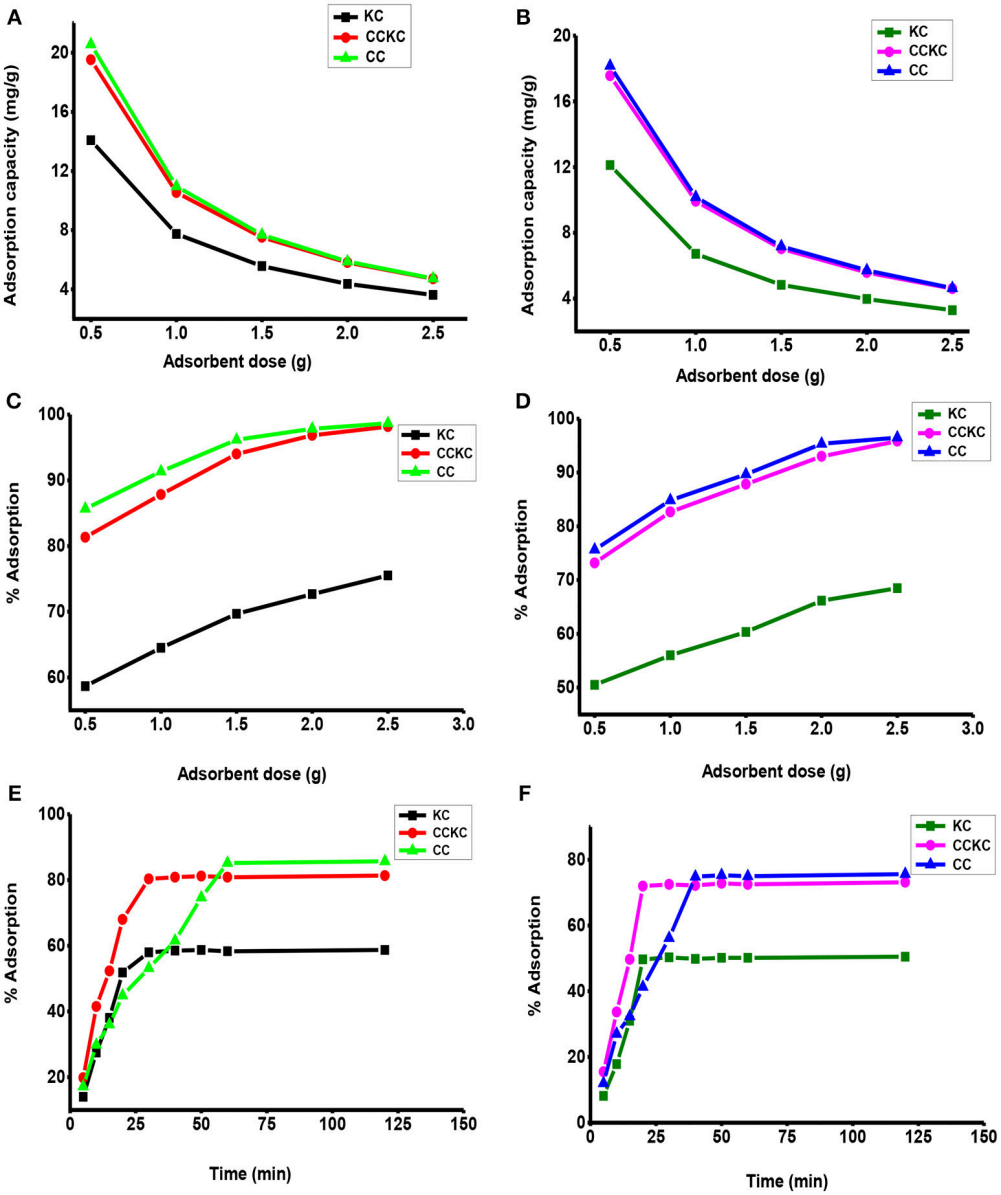
Effect of adsorbent dosage on the adsorption capacity of the adsorbents for **(A)** lead **(B)** cadmium and on the percentage removal of **(C)** lead, **(D)** cadmium, effect of contact time on the percentage adsorption of **(E)** lead and **(F)** cadmium ion.

Adsorbent dosage is also a very important parameter which can affect significantly the adsorption of heavy metals on adsorbents (Li et al., 2011). The effect of adsorbent dosage on removal of Pb (II) and Cd (II) is shown in Figure 5. Increase in the percentage adsorption of both metal ions with increase in adsorbent dose was obtained for all the adsorbents. With increase in dosage from 0.5 to 2.5 g an increase in adsorption of Pb (II) from 58.67 to 75.50, 81.33 to 98.17, and 85.67 to 98.67% was obtained for KC, CCKC, and CC respectively. For Cd (II) ions on the respective adsorbents, a similar increase from 50.5 to 68.59, 73.17 to 95.83, and 75.67 to 96.50% was obtained. The increase in adsorption with dosage increase may be attributed to an increase in the number of active sites available (Akpomie and Dawodu, 2016). Similar results have been reported (Njoya et al., 2006; Barka et al., 2013; Islam et al., 2017). Furthermore, a decrease in the adsorption capacity of metal ions with increase in adsorbent dosage on all the adsorbents was obtained as shown in Figure 5. As the adsorbent dosage increased from 0.5 to 2.5 g, the adsorption uptake of Pb (II) ions decreased from 14.08 to 3.62, 19.52 to 4.712, and 20.56 to 4.74 mg/g for KC, CCKC, and CC respectively. Similarly, that of Cd (II) showed a decrease from 12.2 to 3.29, 17.56 to 4.60, and 18.16 to 4.63 mg/g for the respective adsorbents. This decrease in equilibrium adsorption capacity may be due to the higher adsorbent dosage making available more active sites, as a result the sites remaining unused during the sequestration reaction (Li et al., 2011). This decrease can also be attributed to a decrease in the total adsorption surface area and increase in diffusion path length resulting from overlapping or aggregation of sorption sites (Ahluwalia and Goyal, 2005). Similar trends of percentage removal and adsorption uptake have been reported (Miranda et al., 2010; Nessim et al., 2014). The adsorption trend of metal ions on the adsorbents was also in the order CC>CCKC>CC, while that for metal ions was Pb>Cd. The adsorbent dose of 0.5 g was chosen in this study due to its high adsorption capacity for metal ions recorded. The use of large adsorbate amount in column experiments helps in the evaluation of adsorbents maximum uptake capacity (Zafar et al., 2006).

### Influence of contact time

The influence of contact time on the adsorption of metal ion on any adsorbent is important in order to envisage when equilibrium adsorption is achieved. The effect of contact time on the adsorption of Pb (II) and Cd (II) ions unto the adsorbents was determined as shown in Figure 5. An initial rapid sorption was observed which reduced until equilibrium attainment. The presence of vacant sites initially accounted for the fast removal which became saturated as time progressed thereby attaining equilibrium (Quek et al., 1998). It was observed that different equilibrium times were observed for the two metal ions on all the adsorbents. The equilibrium percentage adsorption of Pb (II) ions was 80.33% (CCKC), 58.5% (KC), and 85.17% (CC) at contact time of 30, 40, and 60 min, respectively. Those for the adsorption of Cd (II) ions reached equilibrium at contact times of 20, 30, and 50 min and were 72% (CCKC), 50.33% (KC), and 75.33% (CC), respectively. A contact time of 120 min was utilized to ensure equilibrium sorption of both metal ions on all the adsorbents was attained. The faster rate of adsorption of Cd (II) when compared to Pb (II) is due to the smaller ionic radii of Cd (II) (0.097 nm) than Pb (II) (0.12 nm) which makes its diffusion faster to the surface of adsorbents due to its smaller size than the bulkier Pb (II) ion. Another important observation is that equilibrium was achieved faster for Cd and Pb ions adsorption on CCKC (20, 30 min), followed by KC (30, 40 min), and then CC (50, 60 min). An effective adsorbent for adsorption should not only have a high adsorption capacity but also a faster rate of removal (Madhukar and Mahajan, 2011), this suggest the effectiveness of CCKC over CC and KC for metal ions sorption considering the fact that CC only has a slightly higher adsorption than CCKC. This reveals the potential of this new adsorbent for effective sorption of metal ions from solution.

### Kinetic model analysis

Kinetic modeling evaluates adsorption mechanism, which include mass transfer, chemical reaction and diffusion control (Farah and El-Gendy, 2013). The pseudo first order (PFO), pseudo second order (PSO), intraparticle diffusion (ID), and liquid film diffusion (LFD) rate equations were utilized. The kinetic rate constants obtained from the application of the kinetic model equation for the adsorption of Pb (II) and Cd (II) ions unto the adsorbents are shown in Table 4. From the PFO *R*^2^ values obtained, it was observed that this model did not show good fit to the adsorption; as the values were lower than 0.9. Except for the sorption of Pb (II) ion on KC where the *R*^2^ was as high as 0.975 but still lower than that of PSO model. The values of the *qe*_*exp*_ also showed great discrepancy with the *qe*_*cal*_ of the PFO model for both metal ions on the adsorbents. The low *R*^2^ values and difference between the *qe*_*cal*_ and *qe*_*exp*_ simply showed that sorption on KC, CCKC and CC did not follow the PFO kinetic mechanism. It was observed from the values of the linear regression coefficient (*R*^2^) that the PSO model presented a good and best fit to the sorption on all the adsorbents. Also the values of *qe*_*cal*_ were closer to the values of *qe*_*exp*_ than those presented by the PFO model. Similar results have been reported (Das and Mondal, 2011; Barka et al., 2013; Meitei and Prasad, 2013). Effective evaluation of sorption mechanism could be arrived at after thermodynamics and desorption performance are considered (Akpomie et al., 2017).

**Table 4:** Kinetic model constants for the adsorption of Pb (II) and Cd (II) on the adsorbents.

		**Pb (II)**			**Cd (II)**	
**Model/Adsorbent**	**KC**	**CCKC**	**CC**	**KC**	**CCKC**	**CC**
qe_exp_ (mg/g)	14.08	19.52	20.56	12.12	17.56	18.16
**PFO MODEL**						
qe_cal_ (mg/g)	39.7	21.8	37.4	7.3	10	39.7
K_I_ (min^−I^)	0.17	0.11	0.07	0.09	0.09	0.10
*R*^2^	0.975	0.867	0.746	0.659	0.714	0.876
**PSO MODEL**						
h (mg/g min)	1.64	2.34	0.96	0.98	2.02	0.95
K_2_ (g/mg min)	6.5 × 10^−3^	4.7 × 10^−3^	1.4 × 10^−3^	4.7 × 10^−3^	5.04 × 10^−3^	1.7 × 10^−3^
qe_cal_ (mg/g)	15.87	22.22	26.32	14.49	20	23.81
*R*^2^	0.979	0.983	0.980	0.935	0.971	0.946
**ID MODEL**						
K_d_ (mg/g min^1/2^)	1.168	1.598	2.075	1.107	1.431	1.955
C	4.6	6.5	1.2	3.2	6.1	1.4
*R*^2^	0.611	0.632	0.893	0.555	0.548	0.778
**LFD MODEL**						
K_fd_	0.172	0.11	0.07	0.094	0.09	0.11
D	1.04	0.11	0.6	0.51	0.56	0.78
*R*^2^	0.975	0.867	0.74	0.659	0.714	0.876

Metal ions on adsorbent surfaces may diffuse inwards into the porous surface and thus follow the ID mechanism (Weber and Morris, 1963). The values of *R*^2^ obtained for ID model on the adsorption of both metal ions on all the adsorbents were low (*R*^2^ < 0.894) indicating that ID is not the rate controlling mechanism for the sorption of the metal ions on the adsorbents. Furthermore, the occurrence of the intercept *C*, showed the involvement of surface sorption process (Yadav et al., 2014). If significant sorption is achieved during movement of adsorbate from solution to adsorbent surface, the process would likely conform to the LFD model (Taffarel and Rubio, 2009). As observed, all the *R*^2^ values obtained for LFD for both metal ions were lower than those of the PSO model, but higher than the ID model. This implies that film diffusion (surface phenomenon) played a more dominant role in the sorption process than ID.

### Thermodynamic and desorption analysis

The Δ*G*^0^, Δ*H*,^0^ and Δ*S*^0^ values were calculated to evaluate the feasibility of adsorption. The calculated thermodynamic parameters of adsorption are given in Table 5. The reaction was found to be spontaneous due to negative Δ*G*^0^ values at all temperatures on all the adsorbents. The negative Δ*H*^0^ values obtained for the two metal ions on KC and CC indicated an exothermic adsorption process which was supported by the good fit of these adsorbents to the Langmuir model. Negative values of Δ*S*^0^ suggested that the process is enthalpy driven. On the other hand CCKC showed positive Δ*H*^0^ values for both metal ions indicating an endothermic sorption process. This implies higher temperatures favor the adsorption process on CCKC indicating the adsorbent would be suitable in temperate regions like Nigeria were temperatures could be as high as 30 to 40°C in certain seasons. The heat (Δ*H*^0)^ of physisorption is within the range of 2.1–20.9 kJ/mol, while chemisorptions is within 80–200 kJ/mol (Das and Mondal, 2011). From Table 5, the values of Δ*H*^0^ for KC and CC were high than 20.9 KJ/mol but less than 80 kJ/mol which suggested a physicochemical process rather than a chemical or physical one. Interestingly, adsorption on CCKC showed Δ*H*^0^ values in the physisorption range for both metal ions. This physisorption is desirable in enabling easy desorption of metal ion from the metal loaded adsorbents for reuse and recycling.

**Table 5:** Thermodynamic parameters for the adsorption of Pb (II) and Cd (II) on the adsorbents.

**Parameter/Adsorbent dose**	**KC**	**CCKC**	**CC**
**Pb (II)**			
ΔH^0^ (kJ/mol)	−25.6	14.71	−27.4
ΔS^0^ (J/molK)	−44.3	52.84	−42.8
300K(ΔG^0^)(kJ/mol)	−2.74	−1.62	−3.21
313K(ΔG^0^)(kJ/mol)	−2.51	−1.83	−3.01
323K(ΔG^0^)(kJ/mol)	−2.37	−1.97	−2.93
**Cd (II)**			
ΔH^0^ (kJ/mol)	−22.6	12.81	−24.1
ΔS^0^ (J/molK)	−40.1	48.31	−39.6
300K(ΔG^0^)(kJ/mol)	−2.43	−1.41	−3.04
313K(ΔG^0^)(kJ/mol)	−2.21	−1.57	−2.83
323K(ΔG^0^)(kJ/mol)	−2.03	−1.65	−2.64

The result of desorption of Pb (II) from metal loaded KC, CCKC, and CC showed percentage desorption of 72.6, 81.3, and 70.5% in acid media respectively. That obtained in basic media was 65.7, 74.2, and 61.3% for the respective adsorbents. Cadmium ion desorption from metal loaded KC, CCKC, and CC gave desorption percentages of 70.3, 84.2, and 65.4 in acid media and 56.4, 77.8, 59.3 using 0.1M NaOH as eluent. This indicated the effectiveness of using 0.1M HCl in removal of biosorbed metal ions from the surface of BFSH than using 0.1M NaOH. Several studies have showed HCl to be more effective than other eluents such as HNO_3_, H_2_SO_4_, and NaOH in desorbing metals from adsorbents (Gautam et al., 2014). The highest percentage desorption obtained using both eluent on CCKC for both metal ions indicated the suitability as an efficient adsorbent which can easily be recycled and reused. The highest percentage desorption corroborated the physisorption mechanism obtained for CCKC from the thermodynamic analysis.

## Conclusions

From this research it was discovered that the addition of corn cob to kaolinite clay and calcinations improved its adsorption capacity for lead and cadmium ions. However, the corn cob recorded a higher adsorption than the corn cob-kaolinite composite, suggesting the use of corn cob in improving the adsorption capacity of kaolinite but not vice versa. However, the corn cob-kaolinite composite showed a faster rate of removal of metal ions in the kinetic analysis than the kaolinite clay and corn cob adsorbents which is desirable. This indicated that corn cob could be efficiently utilized in enhancing the adsorption capacity of kaolinite clay for metal ions, producing an adsorbent with higher adsorption and fast metal removal rate. The new corn cob kaolinite combo adsorbent was also found to involve physisorption mechanism and recorded higher desorption of both metal ions in both acidic and basic media which suggest easy recyclability and res-use of the adsorbent. This provides a cheaper alternative means of clay modification rather than the excessively used chemical means which is expensive and noxious to the environment.

## Author contributions

KA and PE designed the research work and carried out the Laboratory experiments along with HC-O. The write up was compiled by KA, PE, and HC-O while kinetic; isotherm, thermodynamic evaluations and the experimental graphs were analyzed and plotted by CO.

### Conflict of interest statement

The authors declare that the research was conducted in the absence of any commercial or financial relationships that could be construed as a potential conflict of interest.
